# Virtual Reality Orthoptic Interventions for Binocular Vision Disorders: A Systematic Review and Meta-Analysis

**DOI:** 10.3390/jemr19020039

**Published:** 2026-04-14

**Authors:** Clara Martinez-Perez, Noelia Nores-Palmas, Jacobo Garcia-Queiruga, Maria J. Giraldez, Eva Yebra-Pimentel

**Affiliations:** 1 Applied Physics Department (Optometry Area), Facultade de Óptica e Optometría, Universidade de Santiago de Compostela, 15705 Santiago de Compostela, Spain; claramartinez.perez@usc.es; 2 GI-2092 Optometry, Departamento de Física Aplicada (Área de Optometría), Universidade de Santiago de Compostela, 15782 Santiago de Compostela, Spain; noelia.nores@rai.usc.es (N.N.-P.);; 3 AC-24 Optometry, Instituto de Investigación Sanitaria de Santiago de Compostela (IDIS), 15706 Santiago de Compostela, Spain

**Keywords:** convergence insufficiency, intermittent exotropia, stereopsis

## Abstract

Purpose: To systematically review and meta-analyze randomized controlled trials (RCTs) evaluating digital orthoptic interventions, including virtual reality (VR)–based approaches, for convergence insufficiency and intermittent exotropia. Methods: This systematic review and meta-analysis followed PRISMA 2020 guidelines and AMSTAR-2 standards and was prospectively registered in PROSPERO. PubMed, Web of Science, and Scopus were searched up to December 2025. Eligible studies were RCTs comparing VR-based or digital orthoptic interventions with conventional therapy, placebo VR, or control conditions. Primary outcomes included near point of convergence, ocular deviation, fusional reserves, and stereopsis. Risk of bias was assessed using RoB 2 and certainty of evidence with GRADE. Results: Four RCTs (184 participants) were included. In convergence insufficiency, digital orthoptic interventions, including VR-based approaches, significantly reduced near heterophoria (mean difference [MD] −1.64 prism diopters; 95% CI −3.17 to −0.12), with no significant effects on near point of convergence or positive fusional reserves. In intermittent exotropia, VR-based interventions significantly improved near point of convergence (MD −1.60 cm; 95% CI −2.64 to −0.55), although this change did not reach the ≥4 cm threshold considered clinically meaningful according to the Convergence Insufficiency Treatment Trial. Improvements were also observed in stereopsis (MD −0.19 log units; 95% CI −0.33 to −0.04), while changes in near deviation were not significant. Evidence certainty ranged from low to moderate. Conclusions: VR-based and digital orthoptic interventions may offer modest, outcome-specific benefits as adjunctive treatments for selected binocular vision disorders. Larger, well-designed RCTs with standardized outcomes are needed.

## 1. Introduction

Virtual reality (VR) is a computer-generated three-dimensional environment that allows users to experience and interact with immersive digital scenarios through head-mounted displays (HMDs) [1,2]. By presenting different images to each eye, VR systems generate a stereoscopic perception of depth that closely simulates real-world visual experiences [3,4,5,6,7,8]. Owing to advances in display resolution, processing speed, and motion tracking, VR technology has rapidly expanded beyond entertainment and gaming, finding applications in education, rehabilitation, and medical and vision-related research [5,6,9].

In vision science, VR has attracted growing interest as a platform for both the assessment and rehabilitation of binocular vision [10,11]. The ability of VR systems to deliver controlled, binocularly dissociated stimuli within immersive environments makes them particularly suitable for targeting vergence, fusion, and stereoscopic processing. Accordingly, VR-based approaches have been explored to support binocular vision assessment and training across a range of clinical conditions, including strabismus and vergence disorders [1,3,5,12,13].

Efforts to digitize classical binocular vision assessments, such as Hess or tangent screen–based tests, have also been reported using objective eye-tracking and computerized approaches [14]. Although these digital adaptations improve standardization and facilitate data recording, they often rely on external projection systems and do not fully exploit the immersive and interactive capabilities inherent to VR environments [15,16,17]. Consequently, despite technological progress, the translation of digital tools into clinically effective orthoptic assessment and training paradigms remains limited, and evidence regarding their therapeutic effectiveness is still emerging [3,12].

Beyond diagnostic applications, VR has gained increasing attention as a potential tool for vision rehabilitation and orthoptic training. Its immersive nature, combined with the capacity to present adaptive and engaging binocular tasks, offers potential advantages for improving functional binocular performance and patient engagement. However, existing studies differ substantially in terms of clinical populations, intervention protocols, outcome measures, and methodological quality, which hampers the interpretation and comparison of findings and precludes definitive conclusions regarding effectiveness [7,8].

From a technological perspective, modern head-mounted VR systems increasingly incorporate integrated eye-tracking modules, enabling objective and real-time recording of ocular behavior within immersive environments. However, most current displays present a fixed accommodative demand while manipulating vergence through binocular disparity, thereby generating a vergence–accommodation conflict that may reduce visual comfort and potentially limit the physiological transfer and effectiveness of VR-based orthoptic therapy [18]. These systems typically employ infrared cameras to estimate gaze position relative to the corneal reflex, allowing continuous monitoring of oculomotor behavior [19,20]. While eye-tracking has shown promise for the objective assessment of ocular alignment, its availability remains limited, and it is not a prerequisite for VR-based orthoptic training, as many current interventions rely on stimulus-driven binocular tasks rather than gaze-contingent feedback [3,12].

Therefore, the objective of the present study was to systematically review and meta-analyze randomized controlled trials (RCTs) evaluating digital orthoptic interventions, including virtual reality-based approaches, for binocular vision disorders, specifically convergence insufficiency and intermittent exotropia, and to quantify their effects on clinically relevant binocular outcomes, including near point of convergence, ocular deviation, fusional reserves, and stereopsis.

## 2. Materials and Methods

### 2.1. Research Question and PICOS Framework

**Figure 1:**
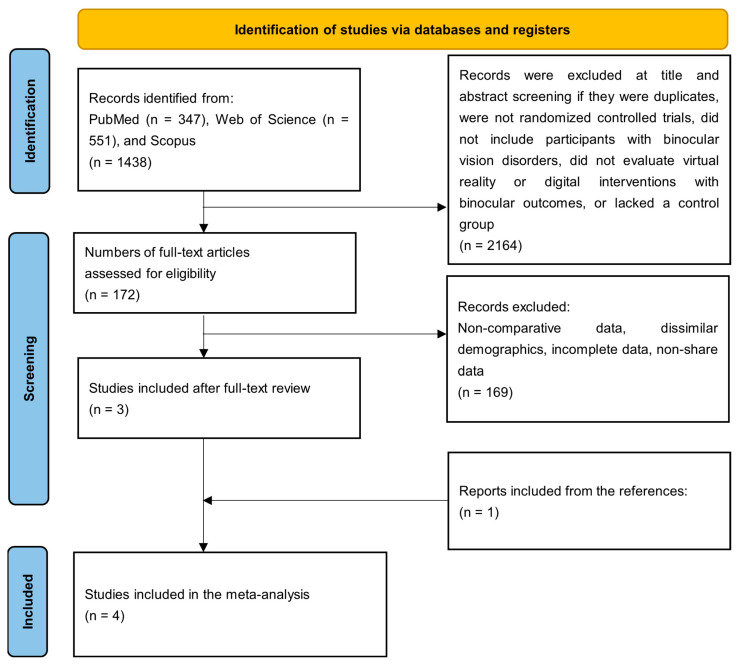
PRISMA flow diagram of study selection.

This systematic review and meta-analysis were conducted in accordance with the PRISMA 2020 [21] guidelines and followed AMSTAR-2 [22] methodological standards (Figure 1). The review protocol was prospectively registered in PROSPERO (registration number: [CRD420261280576]). A completed PRISMA checklist is provided as Supplementary Material File S1. The final literature search was performed on 19 December 2025.

The research question was formulated using the PICOS framework to ensure methodological rigor and clinical relevance. Specifically, the review aimed to evaluate whether individuals with binocular vision disorders, including convergence insufficiency and intermittent exotropia (Population), demonstrate improvements in motor and sensory binocular outcomes (Outcomes) when treated with digital orthoptic interventions, including VR-based approaches (Intervention), compared with conventional orthoptic therapy, placebo virtual reality, or other control conditions (Comparator).

Eligible studies were restricted to randomized controlled trials (RCTs) assessing digital orthoptic interventions, including immersive VR and non-immersive computer- or mobile-based platforms, targeting binocular vision. Primary outcomes were a priori categorized into motor outcomes (near point of convergence, ocular deviation, and positive fusional vergence) and sensory outcomes (stereopsis) to facilitate physiologically meaningful interpretation. Secondary analyses explored differences according to intervention type and clinical condition as potential sources of heterogeneity. By synthesizing the available evidence, this review sought to clarify the role of VR-based approaches in the management of binocular vision disorders and to provide clinically relevant insights to inform practice and future research.

### 2.2. Eligibility Criteria

Studies were excluded if they met any of the following criteria: case reports, case series, conference abstracts, or studies lacking a comparative or control group; narrative reviews, systematic reviews, or meta-analyses; or duplicate publications derived from the same dataset. Risk-of-bias assessments were not used as exclusion criteria; instead, RoB 2 ratings were incorporated into the GRADE evaluation and interpretation of findings. Additional exclusion criteria included insufficient methodological detail, incomplete or non-comparable demographic data, unclear diagnostic definitions of binocular vision disorders, heterogeneous or non-standardized outcome measures, absence of clinically relevant binocular outcomes (e.g., near point of convergence, fusional vergence, ocular deviation, or stereopsis), or insufficient statistical data (e.g., missing sample sizes, means, standard deviations, or effect estimates) required for quantitative synthesis.

### 2.3. Information Sources

A comprehensive literature search was conducted across three major electronic databases: PubMed, Web of Science, and Scopus, without restrictions on publication year or language. The search strategy was designed to identify all RCTs evaluating digital orthoptic interventions, including VR-based approaches, for binocular vision disorders. In addition, the reference lists of all included studies and relevant reviews were manually screened to identify any additional eligible articles not retrieved through the electronic search.

### 2.4. Search Methods for Identification of Studies

The literature search was conducted using a comprehensive strategy combining controlled vocabulary and free-text terms related to binocular vision disorders, including convergence insufficiency, accommodative and vergence dysfunctions, and strabismus, as well as digital or VR–based interventions, such as VR, computer-based and game-based approaches, and digital or objective assessment tools. Key search terms included combinations of “virtual reality”, “eye tracking”, “convergence insufficiency”, “intermittent exotropia”, and “binocular vision”. Full search strategies for each database are provided in Supplementary Material File S2.

Two reviewers independently screened titles and abstracts for eligibility, followed by full-text evaluation of potentially relevant studies. Disagreements regarding study selection or data extraction were resolved by consensus. No restrictions on publication year or language were applied. Studies published in English, Spanish, or Portuguese were assessed directly, while articles in other languages were translated when sufficient data were available for inclusion.

### 2.5. Data Extraction and Data Items

Two reviewers independently extracted data from all eligible studies using a standardized data extraction form. For each included study, the following information was collected: first author and year of publication, country, study design, sample size, participant age, clinical condition (convergence insufficiency or intermittent exotropia), diagnostic criteria. For studies including participants with convergence insufficiency, the number of clinical signs used to define the condition was also recorded.

Characteristics of the intervention and comparator were also extracted. Details related to the digital modality were collected, including type of intervention (VR or computer-based), device used, presence or absence of eye-tracking, and intervention duration. In addition, when reported, the underlying vergence stimulation principles of the digital interventions were extracted. In VR-based interventions, when used, vergence demand was typically generated by presenting dichoptic targets with horizontal disparity between the images shown to each eye, thereby inducing vergence responses. Depending on the intervention design, this stimulation primarily induced convergence demand or a combination of convergence and divergence. In some studies, vergence demand changed progressively during the task (smooth ramp), whereas in others it was modified in discrete steps or incorporated into game mechanics requiring sustained binocular fusion.

Primary outcome measures extracted for quantitative synthesis included near point of convergence, positive fusional vergence, angle of deviation, fusional control scores, and stereopsis, depending on the clinical condition assessed. When available, outcome data were collected for all reported time points. Additional variables, such as follow-up duration, compliance, and reported conflicts of interest, were also recorded to support subgroup analyses and assessment of methodological quality. Any discrepancies in data extraction were resolved through discussion and consensus.

Study record management, including duplicate removal and tracking of inclusion and exclusion decisions, was conducted using Rayyan tool (Rayyan Systems Inc., Qatar Computing Research Institute, Doha, Qatar).

### 2.6. Risk of Bias Assessment

The methodological quality and risk of bias of the included RCTs were independently assessed by two reviewers using the Cochrane Collaboration’s Risk of Bias tool (RoB 2; The Cochrane Collaboration, Oxford, UK). This tool evaluates potential sources of bias across the following domains: bias arising from the randomization process, deviations from intended interventions (performance bias), bias due to missing outcome data (attrition bias), bias in measurement of the outcome (detection bias), and bias in selection of the reported results (reporting bias).

**Figure 2:**
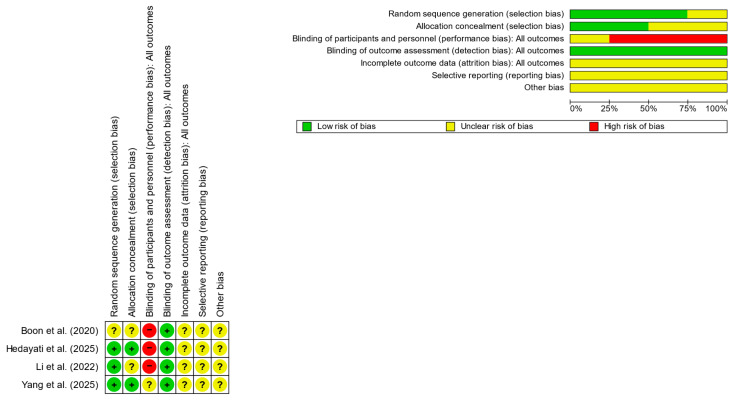
Risk of bias assessment (green = low risk; red = high risk; yellow = unknown) of 4 RCTs [23,24,25,26].

Each domain was judged as having low risk of bias, some concerns, or high risk of bias according to the RoB 2 guidance. Any discrepancies between reviewers were resolved through discussion and consensus. The overall results of the Cochrane risk of bias assessment are illustrated in Figure 2, with detailed domain-specific justifications provided in Supplementary Material File S3. Risk-of-bias judgments were not used to exclude studies but were incorporated into GRADE certainty ratings and sensitivity interpretation.

### 2.7. Assessment of Results

All outcomes related to binocular vision and accommodative–vergence function were analyzed quantitatively when sufficient data were available. Dichotomous outcomes were summarized using odds ratios (OR) with 95% confidence intervals (CI), while continuous outcomes obtained from digital or objective assessments were analyzed using mean differences (MD) or standardized mean differences (SMD), as appropriate. When possible, the magnitude of pooled effects was interpreted in relation to commonly accepted clinical benchmarks in binocular vision practice to aid clinical relevance assessment. Mean differences were pooled only when outcome measures were reported in clinically comparable units across studies. For near point of convergence (NPC), measurements were obtained using standard clinical push-up procedures. Although the specific measurement instrument or anatomical reference point (e.g., nasion, orbital rim, or nose tip) was not always explicitly reported across studies, NPC values were consistently expressed in centimeters. Therefore, these measurements were considered clinically comparable for quantitative synthesis.

Statistical heterogeneity was assessed using the I^2^ statistic and interpreted as low (<25%), moderate (25–50%), or high (>50%). Fixed-effects models were applied when heterogeneity was low (I^2^ ≤ 50%), and random-effects models were used when substantial heterogeneity was present. Missing or incomplete data were handled according to recommendations from the Cochrane Handbook [27]. All meta-analyses were performed using Review Manager (RevMan) version 5.4.1 (The Cochrane Collaboration, London, UK).

### 2.8. Publication Bias

Potential publication bias was explored descriptively using funnel plots generated in RevMan v5.4 software (Copenhagen, Denmark). However, given the small number of included studies per outcome (<10), formal assessment of small-study effects is inherently underpowered and funnel plot findings were interpreted cautiously.

### 2.9. Additional Analyses

When data permitted, subgroup analyses were conducted according to participant age, type of binocular dysfunction, and digital assessment modality. The certainty of evidence for each outcome was assessed using the GRADE framework [28], considering risk of bias, inconsistency, indirectness, imprecision, and publication bias. Outcomes were classified as very low, low, moderate, or high certainty of evidence. All assessments were conducted independently by two reviewers, with disagreements resolved through discussion.

## 3. Results

### 3.1. Study Selection

A total of 2336 records (Figure 1) were identified from PubMed (n = 347), Web of Science (n = 551), and Scopus (n = 1438). After title and abstract screening and duplicate removal, 2164 records were excluded. A total of 172 full-text articles were assessed for eligibility, of which 169 were excluded due to non-comparative study designs, non-relevant populations, insufficient outcome data, or other eligibility criteria not being met. Three studies met the eligibility criteria, and one additional study was identified through reference screening, resulting in four studies included in the meta-analysis [23,24,25,26].

### 3.2. Study Characteristics

Table 1 summarizes the main characteristics of the four studies included in this review, all evaluating digital orthoptic interventions, including immersive virtual reality–based and non-immersive computer- or mobile-based approaches, for binocular vision disorders. The studies were conducted in China, Australia, South Korea, and Iran, using randomized designs with sample sizes ranging from 18 to 63 participants. Two studies assessed convergence insufficiency without manifest strabismus, while two focused on intermittent exotropia. In the studies evaluating convergence insufficiency, diagnostic criteria were based on standard clinical signs, including near exophoria greater than distance exophoria, receded near point of convergence, and reduced positive fusional vergence. Boon et al. [23] used a three-sign diagnostic definition, whereas Li et al. [25] required at least two of the three diagnostic signs. No eligible randomized controlled trials were identified for other binocular vision anomalies despite the comprehensive and intentionally broad search strategy across multiple databases and without language or date restrictions, indicating a current gap in the randomized evidence rather than a restriction of the review scope or methodology. Interventions included VR–based training, gamified VR, and computer- or mobile-based dichoptic therapies, compared with conventional orthoptic approaches, placebo VR, or patching. The vergence stimulation principles differed across the VR-based interventions. In Boon et al. [23], vergence demand was manipulated dynamically within a gamified VR environment by adjusting the angular separation between dichoptic images, allowing both convergence and divergence training through continuously changing disparity levels integrated into game mechanics. In Li et al. [25], vergence demand was generated using horizontally separated dichoptic targets, with prismatic demand ranging from approximately 20 prism diopters base-in to 30 prism diopters base-out and increasing progressively during training (smooth ramp stimulation). In Yang et al. [26], the VR game was designed primarily to stimulate convergence (base-out demand) through interactive tasks requiring sustained binocular fusion. In contrast, Hedayati et al. [24] used a non-immersive dichoptic video game (PIVOT) combined with conventional orthoptic therapy rather than an immersive VR system to promote binocular fusion. Primary outcomes included near point of convergence, positive fusional vergence, angle of deviation, fusional control, and stereopsis, with follow-up periods from 4 weeks to 6 months. Heterogeneity in populations, interventions, and outcome measures was considered in the data synthesis.

### 3.3. Outcomes

Outcomes were analyzed according to their physiological domain, distinguishing between motor binocular outcomes (e.g., heterophoria, near point of convergence, deviation) and sensory outcomes (stereopsis).

**Figure 3:**
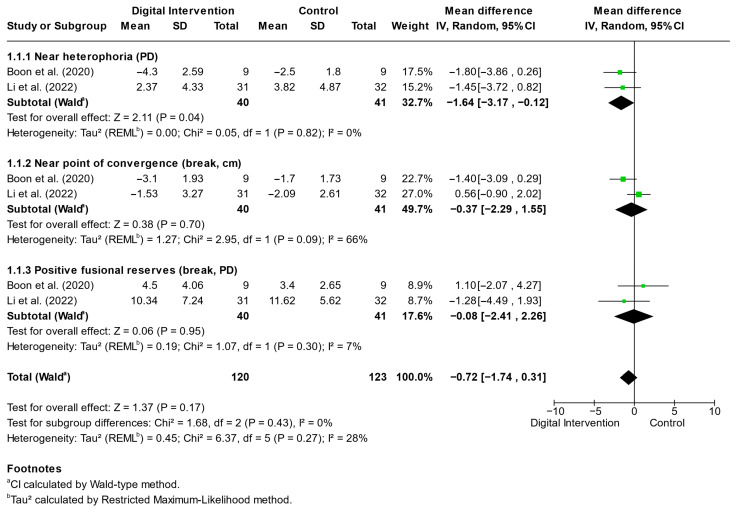
Forest Plot Showing the Effects of Digital Orthoptic Interventions on Clinical Outcomes in Convergence Insufficiency. Pooled estimates are based on two studies per outcome and should be interpreted with caution given the limited evidence base [23,25].

Figure 3 illustrates the meta-analytic comparison between digital orthoptic interventions and control conditions for convergence insufficiency. Digital orthoptic interventions were associated with a small but statistically significant improvement in near heterophoria compared with controls (*p* = 0.040). However, the magnitude of change (MD −1.64Δ) was modest and likely near or below commonly used clinical relevance thresholds in practice, where differences of approximately ≥3–4 prism diopters are often considered more clinically meaningful [29]. No significant effects were observed for near point of convergence or positive fusional reserves (both *p* ≥ 0.700). Overall heterogeneity was low to moderate (I^2^ = 28%).

Li et al. [25] reported the Convergence Insufficiency Symptom Survey (CISS). In that trial, mean CISS scores decreased from 27.69 ± 8.50 to 21.75 ± 9.13 in the VR group and from 25.18 ± 9.40 to 16.47 ± 8.02 in the OBVAT group at 12 weeks, indicating significant within-group improvement over time (*p* < 0.001) but no significant between-group difference (*p* = 0.514); therefore, quantitative pooling of symptom outcomes was not feasible. These findings suggest that digital orthoptic interventions, particularly immersive VR approaches, may preferentially improve near motor alignment, whereas effects on convergence capacity remain inconsistent across studies.

**Figure 4:**
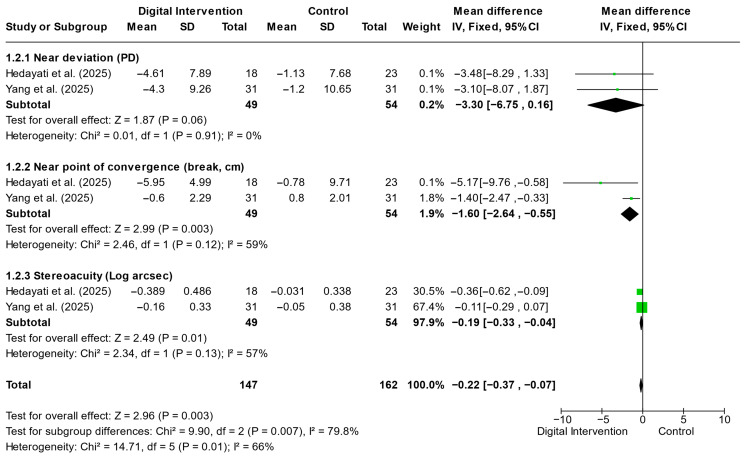
Forest Plot Showing the Effects of Digital Orthoptic Interventions on Clinical Outcomes in Intermittent Exotropia. Pooled estimates are based on two studies per outcome and should be interpreted with caution given the limited evidence base [24,26].

Figure 4 summarizes the meta-analytic comparison between digital orthoptic interventions and control conditions in patients with intermittent exotropia. The pooled analysis indicated statistically significant improvements in near point of convergence and stereopsis (both *p* ≤ 0.010), while changes in near deviation approached but did not reach statistical significance (*p* = 0.060). Nevertheless, the absolute magnitude of change was relatively small, and the clinical significance of these improvements should be interpreted cautiously. Moderate heterogeneity was observed across outcomes, reflecting variability in intervention protocols and participant characteristics. Both included intermittent exotropia trials reported fusional control outcomes. In Yang et al. [26], the Newcastle Control Score improved from 4.3 ± 1.9 to 3.5 ± 2.0 at 4 weeks (*p* = 0.001) and the Office Control Score from 4.1 ± 2.3 to 3.3 ± 2.4 (*p* = 0.003) in the VR group. Hedayati et al. [24] also reported significant improvements in distance and near control scores in the intervention arms. However, due to the use of different control scales and reporting formats, quantitative pooling was not feasible. Overall, the findings support a potential adjunctive role of digital orthoptic training, although the strength of clinical impact appears limited.

### 3.4. Publication Bias

**Figure 5:**
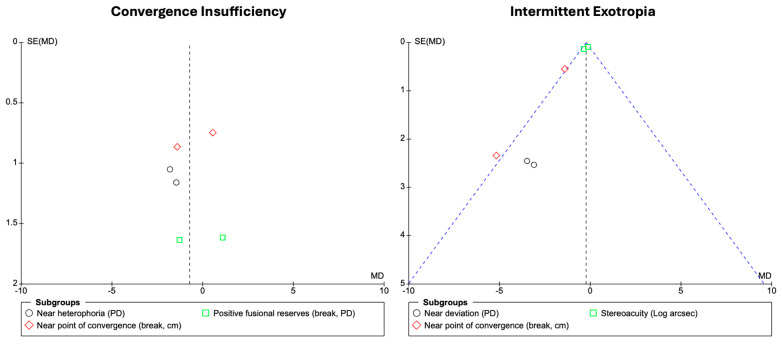
Assessment of publication bias.

Publication bias was explored visually using funnel plots for convergence insufficiency and intermittent exotropia (Figure 5). Given the small number of studies available per outcome, the interpretability of funnel plot symmetry is inherently limited. Therefore, these analyses should be considered exploratory, and no firm conclusions regarding publication bias can be drawn.

### 3.5. GRADE

The GRADE (Grading of Recommendations, Assessment, Development and Evaluation) summary of findings is presented in Table 2. For convergence insufficiency, the certainty of evidence ranged from moderate to low across outcomes. Certainty was rated as moderate for near heterophoria and positive fusional reserves, while near point of convergence was downgraded to low certainty due to inconsistency between studies and imprecision related to the limited number of trials and small sample sizes. For intermittent exotropia, the certainty of evidence was judged as moderate for all evaluated outcomes. Although all included studies were randomized controlled trials with low risk of bias, the certainty of evidence was downgraded due to imprecision arising from the small number of trials and participants. All outcomes were considered critical for clinical decision-making.

## 4. Discussion

This systematic review and meta-analysis synthesized evidence from randomized controlled trials evaluating digital orthoptic interventions, including VR-based approaches, for convergence insufficiency and intermittent exotropia. Overall, the findings indicate that digital orthoptic interventions may provide modest but statistically significant improvements in selected binocular outcomes, particularly near heterophoria in convergence insufficiency (Figure 3) and near point of convergence and stereopsis in intermittent exotropia (Figure 4), when compared with conventional therapy or placebo digital conditions. However, these effects were outcome-specific and heterogeneous, with inconsistent findings for fusional reserves and convergence capacity.

The present findings are broadly consistent with evidence from external interventional and experimental studies exploring binocular function in VR environments. Calderón-González et al. [30] reported that a short, individualized VR-based perceptual learning protocol led to improvements in stereoacuity and overall binocular function in adults with severe stereodeficiency, with partial transfer to clinical measures such as positive fusional vergence. Although that study was not randomized and primarily targeted perceptual learning rather than vergence therapy, the observed gains in stereopsis are consistent with the improvements identified in intermittent exotropia in the present meta-analysis. In contrast, experimental work by McAnally et al. [31] demonstrated that immersive VR viewing is associated with systematic vergence errors that may exceed Panum’s fusional range, often occurring without conscious diplopia and persisting even when the vergence–accommodation conflict is optically minimized. These findings suggest that VR environments may differentially affect sensory fusion and motor alignment while limiting consistent improvements in fusional reserves or convergence amplitudes. Importantly, the inherent vergence–accommodation conflict present in most current head-mounted VR displays represents a structural limitation that may constrain the physiological transfer of vergence training effects to natural viewing conditions. This mismatch between vergence demand and accommodative stimulus may partly explain the selective and heterogeneous treatment effects observed across studies and underscores the need for cautious clinical interpretation of VR-based orthoptic outcomes.

A recently published systematic review and meta-analysis by Islam and Dutta Roy [32] also reported beneficial effects of VR-based therapy for convergence insufficiency. While their conclusions are directionally consistent with the present findings, differences in effect size may reflect methodological factors, as their analysis primarily synthesized pre–post changes and included heterogeneous study designs. In contrast, the present meta-analysis was restricted to randomized controlled trials with explicit comparator groups and incorporated formal risk-of-bias and GRADE assessments, yielding a more conservative and clinically interpretable estimate of treatment effects.

In addition, studies examining immersive VR during physical activity, such as those by Christ et al. [33], have reported improvements in oculomotor comfort despite concerns regarding motion sickness or disorientation. Together, these findings support the notion that VR exposure does not necessarily exacerbate oculomotor strain and may facilitate adaptation in certain domains, while simultaneously imposing constraints on precise vergence control. This duality is reflected in the present meta-analysis, which identified selective improvements rather than uniform enhancement across all binocular outcomes.

Although none of the randomized controlled trials included in this meta-analysis incorporated eye-tracking-driven feedback, a growing body of literature supports the feasibility and clinical relevance of eye-tracking within VR environments. Systematic reviews by González-Vides et al. [34] and surveys by Moreno-Arjonilla et al. [20] document the rapid expansion of eye-tracking applications in optometry and VR, highlighting their potential to provide objective, high-resolution measurements of oculomotor behavior.

Validation studies provide important context for interpreting the present findings. Wenner et al. [12] demonstrated good agreement between a head-mounted, immersive VR–based Harms tangent screen test and the conventional method, alongside reduced examination time and automated documentation; however, a systematic shift toward increased esodeviation was observed, likely reflecting higher vergence demand in VR environments. Similarly, Yeh et al. [4] reported near-excellent correlations between immersive VR-based, eye-tracking-assisted measurements of ocular deviation and the alternative prism cover test, while identifying direction-dependent differences, with overestimation of esotropic deviations and underestimation of exotropic deviations. Taken together, these studies suggest that VR-based measurements, while clinically promising, tend to introduce a net esotropic bias related to display optics, accommodation–vergence conflict, and binocular dissociation, which should be considered when interpreting both diagnostic and therapeutic outcomes.

From a clinical perspective, the present findings suggest that digital orthoptic interventions, including VR-based approaches, may serve as a complementary or adjunctive option, rather than a replacement for conventional therapy, in selected patients with convergence insufficiency or intermittent exotropia. Potential advantages include enhanced patient engagement, standardized stimulus delivery, and the feasibility of home-based or hybrid training models, which may improve adherence. However, given the selective nature of observed benefits and the mechanistic constraints of vergence control in VR, clinicians should interpret these results cautiously and avoid extrapolating them to routine first-line clinical practice.

Several limitations should be acknowledged. First, the number of included randomized controlled trials was small (n = 4), limiting statistical power and contributing to imprecision in several pooled estimates. Nevertheless, synthesizing the currently available randomized evidence is important to identify research gaps and inform the design of future trials in this emerging field. Importantly, the restriction to convergence insufficiency and intermittent exotropia reflects the current availability of randomized evidence in this emerging field rather than a limitation imposed by the review design or search strategy. Despite the comprehensive search and absence of language or date restrictions, few controlled trials have evaluated digital or VR-based orthoptic interventions using standardized binocular outcomes. Although all included studies were judged to have low risk of bias according to RoB 2, heterogeneity in intervention protocols, diagnostic criteria, outcome measures, participant characteristics, and follow-up duration may restrict the generalizability of the findings. In addition, while convergence insufficiency and intermittent exotropia share key binocular motor mechanisms, they represent distinct clinical entities with potentially different therapeutic responses; therefore, treatment effects may not be directly comparable despite the stratified analyses performed. Nevertheless, confidence intervals remained relatively wide for several outcomes, reflecting residual imprecision and reinforcing the need for larger, adequately powered, multicenter randomized controlled trials using standardized protocols and clinically meaningful endpoints.

Future research should prioritize adequately powered, multicenter randomized controlled trials comparing digital orthoptic interventions, including VR-based systems, with established orthoptic therapies, using standardized diagnostic criteria and outcome measures. Harmonization of key endpoints, including near point of convergence, fusional reserves, ocular deviation, stereopsis, and symptom-based questionnaires, would facilitate more robust comparisons. Integration of eye-tracking technology within VR platforms represents a critical avenue to enable adaptive, gaze-contingent training and improve mechanistic understanding of treatment response. Longer follow-up periods are needed to assess the durability of effects, alongside evaluation of patient-centered outcomes such as visual symptoms, quality of life, adherence, and usability in home-based or hybrid care models.

## 5. Conclusions

This systematic review and meta-analysis examined randomized controlled trials evaluating digital orthoptic interventions, including VR-based approaches, for convergence insufficiency and intermittent exotropia. Only four trials met the eligibility criteria, highlighting the limited evidence base. Within this context, digital orthoptic interventions were associated with small, outcome-specific improvements, including modest effects on near heterophoria in convergence insufficiency and improvements in near point of convergence and stereopsis in intermittent exotropia, while other outcomes showed inconsistent results. The certainty of evidence ranged from low to moderate due to imprecision and small sample sizes. Overall, digital orthoptic interventions may have a potential adjunctive role, but current evidence is insufficient to support routine clinical implementation, emphasizing the need for larger, well-designed randomized controlled trials.

## Figures and Tables

**Table 1:** Baseline characteristics of the 4 included studies.

Author (Year)	Country	Design	N	Age (Years)	Binocular Disorder	Digital Modality	Device/Hz	Follow-Up	COI
Boon et al. (2020) [23]	Australia	RCT	18	20.8 ± 1.8	Convergence insufficiency	Gamified VR training (Snake Game) vs. anaglyph-based training	Oculus Rift DK2; Hz not reported; no eye-tracking	6 weeks	No
Hedayati et al. (2025) [24]	Iran	RCT	53	10.15 ± 3.80	Intermittent exotropia	Office-based + home-based orthoptic training ± video game (PIVOT) vs. part-time patching	Synoptophore (Takagi) + mobile dichoptic game (PIVOT; non-immersive, non-VR; no eye-tracking); Hz not reported	6 weeks 12 weeks 24 months	No
Li et al. (2022) [25]	China	RCT	63	23.5 ± 1.9	Convergence insufficiency	VR-based vision therapy vs. OBVAT	VR headset; 120 Hz; no eye-tracking	6 weeks 12 weeks	No
Yang et al. (2025) [26]	South Korea	RCT	62	18.1 ± 5.4	Intermittent exotropia	VR head-mounted display orthoptic game vs. placebo VR game	Oculus Go HMD; 60–72 Hz; no eye-tracking	4 weeks (+4-week washout)	No

COI: conflict of interest; HMD: head-mounted display; Hz: hertz; N: sample size; OBVAT: office-based vergence and accommodative therapy; PIVOT: binocular vision stimulation software used for orthoptic training; RCT: randomized controlled trial; VR: virtual reality.

**Table 2:** GRADE assessment of the quality of the evidence and the strength of the recommendations.

Certainty Assessment							Effect	Certainty	Importance
№ of Studies	Study Design	Risk of Bias	Inconsistency	Indirectness	Imprecision	Other Considerations	Mean Difference (95% CI)		
Convergence insufficiency: Near heterophoria									
2	randomized trials	not serious	not serious	not serious	serious ^a^	none	MD − 1.64 (−3.17 to −0.12)	⨁⨁⨁◯ Moderate ^a^	CRITICAL
Convergence insufficiency: Near point of convergence									
2	randomized trials	not serious	serious ^b^	not serious	serious ^a^	none	MD − 0.37 (−2.29 to 1.55)	⨁⨁◯◯ Low ^a,b^	CRITICAL
Convergence insufficiency: Positive fusional reserves									
2	randomized trials	not serious	not serious	not serious	serious ^a^	none	MD − 0.08 (−2.41 to 2.26)	⨁⨁⨁◯ Moderate ^a^	CRITICAL
Intermittent exotropia: Near deviation									
2	randomized trials	not serious	not serious	not serious	serious ^a^	none	MD − 3.30 (−6.75 to 0.16)	⨁⨁⨁◯ Moderate ^a^	CRITICAL
Intermittent exotropia: Near point of convergence									
2	randomized trials	not serious	not serious	not serious	serious ^a^	none	MD − 1.60 (−2.64 to −0.55)	⨁⨁⨁◯ Moderate ^a^	CRITICAL
Intermittent exotropia: Stereoacuity									
2	randomized trials	not serious	not serious	not serious	serious ^a^	none	MD − 0.19 (−0.33 to −0.04)	⨁⨁⨁◯ Moderate ^a^	CRITICAL

CI: confidence interval; MD: mean difference; ⨁ represent the strength of evidence (⨁ very low, ⨁⨁ low, ⨁⨁⨁ moderate, ⨁⨁⨁⨁ high) ^a^ Downgraded due to small sample size and limited number of randomized controlled trials, resulting in wide confidence intervals; ^b^ Downgraded one level for serious inconsistency due to substantial heterogeneity (I^2^ = 66%) and opposite directions of effect between studies, and one level for imprecision due to small sample size and wide confidence intervals.

## Data Availability

The original contributions presented in this study are included in the article/Supplementary Materials. Further inquiries can be directed to the corresponding author.

## Supplementary Materials

The following supporting information can be downloaded at: https://www.mdpi.com/article/10.3390/jemr19020039/s1. Supplementary File S1: PRISMA 2020 checklist; Supplementary File S2: Full electronic database search strategies (PubMed, Web of Science, Scopus); Supplementary File S3: Risk of bias judgment.
