# Supplementary material for: Virtual Reality Orthoptic Interventions for Binocular Vision Disorders: A Systematic Review and Meta-Analysis
Source: J Eye Mov Res. 2026 Apr 14;19(2):39. doi: 10.3390/jemr19020039 (PMC13117401; doi:10.3390/jemr19020039)
Supplement: Supplementary file 1 [file jemr-19-00039-s001.zip › jemr-4113606-Supplementary Material 2-edited.pdf]

Supplementary Material 2. Full electronic search strategies.

Pubmed

Search: **("binocular vision"[Title/Abstract] OR "binocular dysfunction"[Title/Abstract] OR "convergence insufficiency"[Title/Abstract] OR "vergence dysfunction"[Title/Abstract] OR "accommodative dysfunction"[Title/Abstract] OR strabismus[Title/Abstract] OR strabismic[Title/Abstract] OR exotropia[Title/Abstract] OR esotropia[Title/Abstract] OR exodeviation[Title/Abstract] OR esodeviation[Title/Abstract] OR "intermittent exotropia"[Title/Abstract] OR "congenital esotropia"[Title/Abstract]) AND ("eye tracking"[Title/Abstract] OR eyetracking[Title/Abstract] OR "digital assessment"[Title/Abstract] OR "objective measurement"[Title/Abstract] OR "virtual reality"[Title/Abstract] OR videogame*[Title/Abstract] OR "video game*"[Title/Abstract] OR "computer-based"[Title/Abstract] OR computerized[Title/Abstract] OR "screen-based"[Title/Abstract] OR "digital game*"[Title/Abstract])**

("binocular vision"[Title/Abstract] OR "binocular dysfunction"[Title/Abstract] OR "convergence insufficiency"[Title/Abstract] OR "vergence dysfunction"[Title/Abstract] OR "accommodative dysfunction"[Title/Abstract] OR "strabismus"[Title/Abstract] OR "strabismic"[Title/Abstract] OR "exotropia"[Title/Abstract] OR "esotropia"[Title/Abstract] OR "exodeviation"[Title/Abstract] OR "esodeviation"[Title/Abstract] OR "intermittent exotropia"[Title/Abstract] OR "congenital esotropia"[Title/Abstract]) AND ("eye tracking"[Title/Abstract] OR "eyetracking"[Title/Abstract] OR "digital assessment"[Title/Abstract] OR "objective measurement"[Title/Abstract] OR "virtual reality"[Title/Abstract] OR "videogame*"[Title/Abstract] OR "video game*"[Title/Abstract] OR "computer-based"[Title/Abstract] OR "computerized"[Title/Abstract] OR "screen-based"[Title/Abstract] OR "digital game*"[Title/Abstract])

Web of Science

TI=("binocular vision" OR "binocular dysfunction" OR "convergence insufficiency" OR "vergence dysfunction" OR "accommodative dysfunction" OR strabismus OR strabismic OR exotropia OR esotropia OR exodeviation OR esodeviation OR "intermittent exotropia" OR "congenital esotropia") OR AB=("binocular vision" OR "binocular dysfunction" OR "convergence insufficiency" OR "vergence dysfunction" OR "accommodative dysfunction" OR strabismus OR strabismic OR exotropia OR esotropia OR exodeviation OR esodeviation OR "intermittent exotropia" OR "congenital esotropia")) AND (TI=("eye tracking" OR eyetracking OR "digital assessment" OR "objective measurement" OR "virtual reality" OR videogame* OR "video game*" OR "computer-based" OR computerized OR "screen-based" OR "digital game*") OR AB=("eye tracking" OR eyetracking OR "digital assessment" OR "objective measurement" OR "virtual reality" OR videogame* OR "video game*" OR "computer-based" OR computerized OR "screen-based" OR "digital game*")

Scopus

TITLE-ABS-KEY("binocular vision" OR "binocular dysfunction" OR "convergence insufficiency" OR "vergence dysfunction" OR "accommodative dysfunction" OR strabismus OR strabismic OR exotropia OR esotropia OR exodeviation OR esodeviation OR "intermittent exotropia" OR "congenital esotropia") AND TITLE-ABS-KEY("eye tracking" OR eyetracking OR "digital

assessment" OR "objective measurement" OR "virtual reality" OR videogame* OR "video game*" OR "computer-based" OR computerized OR "screen-based" OR "digital game*")
