# Supplementary material for: Virtual Reality Orthoptic Interventions for Binocular Vision Disorders: A Systematic Review and Meta-Analysis
Source: J Eye Mov Res. 2026 Apr 14;19(2):39. doi: 10.3390/jemr19020039 (PMC13117401; doi:10.3390/jemr19020039)
Supplement: Supplementary file 1 [file jemr-19-00039-s001.zip › jemr-4113606-Supplementary Material 3-edited.pdf]

Supplementary Material 3. Risk of bias judgement.

Boon et al. (2020)

A) Participants were allocated to the intervention groups using a pseudo-random process to ensure equal numbers in each group. The method used to generate the allocation sequence was not fully described.

B) The study does not report any method used to conceal allocation prior to assignment, and participants were recruited and allocated within the same setting.

C) Blinding of participants and personnel was not possible due to the nature of the interventions (virtual reality Snake Game versus anaglyph-based training).

D) Clinical vision measurements at baseline and post-intervention were carried out by a researcher who was masked to the intervention group.

E) All enrolled participants completed the six-week intervention and outcome assessments. However, no intention-to-treat analysis was reported.

F) The study reports prespecified clinical outcomes; however, no publicly available study protocol or trial registration was referenced to confirm the absence of selective reporting.

H) This was a small-sample, single-centre feasibility study with a short follow-up period and no placebo or no-treatment control group, which may limit generalisability.

Hedayati et al. (2025)

A) Block randomization was used with randomly varying block sizes (6 and 9), and participants were randomly assigned to three groups.

B) After generating the random sequence, allocation was concealed using sealed envelopes to prevent foreknowledge of group assignment.

C) Due to differences in treatment modalities (patch therapy versus office- and home-based orthoptic training with or without video game software), blinding of participants was not possible.

D) All examinations were performed by an experienced practitioner who was unaware of the patient's group allocation.

E) Of the 72 randomized participants, 53 completed the treatment and follow-up visits. Reasons for dropout were not fully detailed, and no intention-to-treat analysis was reported.

F) The trial was registered (IRCT20211004052668N1), and major outcomes were reported; however, the study protocol was not publicly available to fully assess selective reporting.

H) This was a single-centre study with a relatively small final sample size per group and heterogeneous intervention intensity, particularly within the office-based therapy protocol.

Li et al. (2022)

A) Participants were randomly allocated to the virtual reality-based vision therapy group or the OBVAT group in a 1:1 ratio using a random allocation sequence generated with the Clinical Trial Management Public Platform.

B) The study reports random allocation but does not describe the method used to conceal the allocation sequence from investigators or participants prior to assignment.

C) Due to the nature of the interventions (virtual reality-based vision therapy versus office-based vergence/accommodative therapy), blinding of participants and personnel was not feasible.

D) Outcome assessments were performed by a masked examiner who was unaware of group allocation.

E) A small proportion of participants were lost to follow-up (seven patients in total). Although completion rates were high (>88%), no intention-to-treat analysis was explicitly reported.

F) The trial was registered, and prespecified outcomes were reported; however, the study protocol was not fully accessible to confirm that all planned outcomes were reported.

H) This was a single-centre pilot randomized controlled trial with a relatively small sample size and no placebo or natural history control group, which may limit generalizability.

Yang et al. (2025)

A) Patients were randomized using a computer-based random number generator with a fixed 1:1 allocation ratio and random block sizes.

B) Randomization schedules were concealed from investigators and participants. A laboratory assistant, not involved in outcome assessment, managed visit scheduling and provided device instructions.

C) The study was described as double-blind, and participants in both groups used similar VR head-mounted displays and games. However, the exercise group received convergence-inducing stimuli while the control group did not, which may have allowed participants to perceive differences between interventions.

D) Outcome assessors were masked to group allocation, and examinations were performed by researchers unaware of the randomization results.

E) Of 88 randomized participants, 26 were excluded due to insufficient VR usage or missed visits. Participants with adherence below 70% were excluded from the analysis, and no intention-to-treat analysis was performed.

F) The study reports all prespecified outcomes; however, trial registration was retrospective, and the original protocol was not available for verification.

H) Participants with less than 70% adherence were excluded from analysis, which may introduce adherence-related bias. The washout period was short (4 weeks), limiting assessment of long-term effects.
